# Laboratory 2000 - The challenge of achieving efficiency and compliance

**DOI:** 10.1155/S1463924601000244

**Published:** 2001

**Authors:** John A. Potter

**Affiliations:** Glaxo Wellcome Worldwide Manufacturing and Supply 1011 North Arendell Avenue Zebulon NC 27597 USA

## Abstract

Significant advances within the field of laboratory automation and instrumentation have greatly benefited the pharmaceutical industry in its quest to discover, develop and monitor the quality of its products. Necessitated by the need for efficiency and greater productivity, faster and more cost-effective means of analyses exist in the form of devices made up of complex electromechanical components, all logically controlled and most with the capability to interface with sophisticated information systems. This benefit does come with a price, a greater responsibility to ensure data quality while complying with increased regulatory requirements. Commitment to this responsibility presents a substantial challenge to scientists and managers throughout the industry. Due diligence must be demonstrated. A comprehensive evaluation of every laboratory system utilized, a solid plan of action for correcting any known deficiencies including upgrades or complete replacement, and an accurate monitoring procedure with the ability to measure progress are all absolute necessities to ensure success. Crossfunctional team effott and communication must transpire with full managerial support. Vendors need to be audited, made aware of any functional or quality inadequacies they possess as well as the pharmaceutical industry's expectation for these shortcomings to be rapidly corrected. Suppliers of these systems should also be encouraged to provide complete ‘off-the-shelf solutions’ to eliminate the need for in-house customization. The requirements for regulatory compliance in today's electronic environment have been well publicized. The players involved are not only listening, but also taking the necessary steps to retain and improve efficiency without sacrificing quality. With the proper measures, planning and action, a highly automated, cost-effective and compliant laboratory operation can become a reality.


Journal of Automated Methods & Management in Chemistry, Vol. 23, No. 6 (November–December 2001) pp. 189–190

### Technical note
### Laboratory 2000—The challenge of achieving efficiency and compliance†

**John A. Potter**

*Glaxo Wellcome, Worldwide Manufacturing and Supply, 1011 North Arendell Avenue, Zebulon, NC 27597, USA*

*Significant advances within the field of laboratory automation and instrumentation have greatly benefited the pharmaceutical industry in its quest to discover, develop and monitor the quality of its products. Necessitated by the need for efficiency and greater productivity, faster and more cost-effective means of analyses exist in the form of devices made up of complex electromechanical components, all logically controlled and most with the capability to interface with sophisticated information systems. This benefit does come with a price, a greater responsibility to ensure data quality while complying with increased regulatory requirements. Commitment to this responsibility presents a substantial challenge to scientists and managers throughout the industry. Due diligence must be demonstrated. A comprehensive evaluation of every laboratory system utilized, a solid plan of action for correcting any known deficiencies including upgrades or complete replacement, and an accurate monitoring procedure with the ability to measure progress are all absolute necessities to ensure success. Crossfunctional team effott and communication must transpire with full managerial support. Vendors need to be audited, made aware of any functional or quality inadequacies they possess as well as the pharmaceutical industry's expectation for these shortcomings to be rapidly corrected. Suppliers of these systems should also be encouraged to provide complete 'off-the-shelf solutions' to eliminate the need for in-house customization. The requirements for regulatory compliance in today's electronic environment have been well publicized. The players involved are not only listening, but also taking the necessary steps to retain and improve efficiency without sacrificing quality. With the proper measures, planning and action, a highly automated, cost-effective and compliant laboratory operation can become a reality.*

### Introduction

Global demand for pharmaceutical products is on a steady increase, thus forcing R&D and quality control laboratories to employ more efficient means of sample processing. The obvious alternative to achieve this objective is through automating as much of the sample's life cycle (figure 1) as possible. Various instrumentation, equipment and information systems specifically designed for these tasks are commercially available and are escalating in popularity on an international scale.

The benefits associated with using this advanced technology are not without barriers. Strategic planning is crucial to orchestrate the considerable amount of consistency required to operate at peak efficiency cross divisional and geographical boundaries while maintaining a steady-state of regulatory compliance.

This technical note was written to advocate a course of action, which incoprorates the components necessary to meet the challenges faced by state-of-the-art laboratories. Although slanted towards the demands of a high throughput quality control operation, due consideration will be given to the teamwork, hardware, software and processes required by all that are involved to achieve success.

### Experimental

A sample's life cycle can be divided into five phrases: receipt, preparation, analysis, data acquisition and processing, and data reporting and archival. Standardized approaches to information sharing infrastructure, ana lytical methodology (including development and transfer tactics), instrument platforms, collaborated validation efforts and common work practices must be created and maintained.

Information sharing is the most critical element needed to obtain this standardized state of operation. Development and quality control groups would be the first to initiate this communication and must include representation from all analogous sites involved. Once collective requirements are identified, they can be put under procedural control and implemented into practice. The next phase would be to share this information with external suppliers and solicit responses to determine whether the organizational expectations can be met. At this point, a quality selection process can be accomplished.

A common or compatible information infrastructure needs to be next on the list of essential components and will play a major role in all the phases of a samples lifecycle. This will enable virtually seamless routing of methods, procedures and data from one site to another as well as providing the means for a central repository of communal information that can be controlled and accessed as needed.

Standardization of the analytical technique and intrument platforms paves the way for smooth and efficient transfers from R&D to quality control laboratories. The resulting methodology can be shared across multiple sites with a minimum amount of validation. Retention of the

† This technical note was initially presented at the ISLAR 2000 Conference and is reproduced here by kind permission of Zymark Corporation.

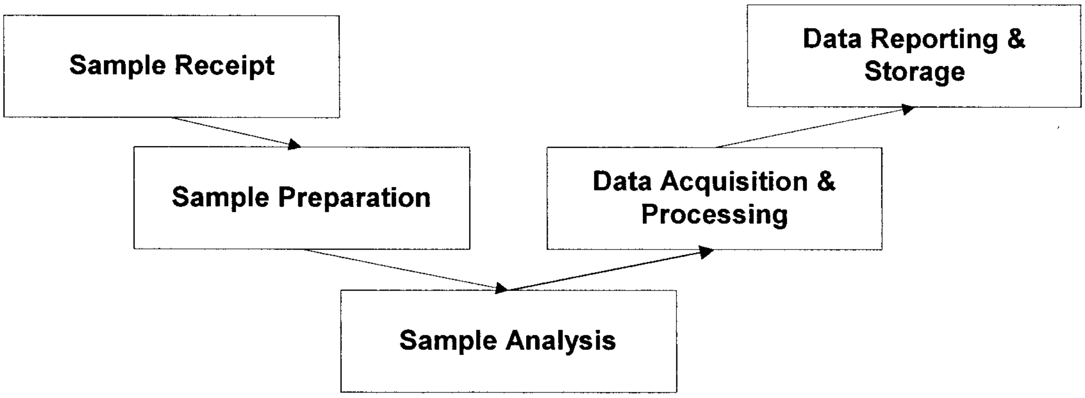

*Figure 1. Sample life cycle.*

technical expertise gained from the development as well as the multiuse attributes associated with this practice stays within the organization. This is of great advantage, especially when employing automated applications that can significantly reduce analytical variance and error when used in a consistent manner.

Once the proper systems are in place, high volume and/or labour-intense tests should be targeted for automated sample preparation and analysis. Examples of these types of tests and laboratory applications are as follows.

*Tests:*

- Assay and impurities.
- Content uniformity.
- Dissolution.
- Gravimetric determination.
- Particle sizing.

*Laboratory applications:*

- Robotics (custom and commercial).
- High-performance liquid chromatographs.
- Gas chromatographs.
- Spectrophotometers (UV/VIS and fluorescence).
- Data-acquisition systems.
- Laboratory information management systems.
- Secured network servers and workstations.

After the samples had been prepared and analysed, the resulting data must be acquired, processed, reported and archived. Substantial care must be given to the treatment of all raw and final data due to the regulatory requirements involved with these phases of a sample's lifecycle. This is where collaborated validation techniques can really pay off in the form of reduced redundancy, and, most importantly, an increase in compliance consistency and quality.

### Results and discussion

After putting the proper communication channels into place, such as face-to-face meetings, shared network directories as well as video- and teleconferencing, the environment is set to begin strategic planning. The 'one team' concept can be implemented, which basically involves R&D and quality control departments exchanging information during the development phase of an analytical method and sharing responsibilities during validation. Emphasis should be placed on automation, which ultimately will improve efficiency. This represents additional challenges in the areas of consistency and validation. Laboratory instrumentation and equipment has to be standardized (identical or proven equivalent). This task can be accomplished by forming technology 'focus groups' empowered to make unified decisions about analytical systems, supplier selection and vali dation strategies. Implementation of this approach combines the expertise of the entire corporation and eventually will yield a compliant and efficient laboratory operation.

### Acknowledgements

The author thanks his colleagues both in the USA and in overseas organizations for their assistance and cooperation.

